# Management strategies and root causes of missed iatrogenic intraoperative ureteral injuries with delayed diagnosis: a retrospective cohort study of 40 cases

**DOI:** 10.1186/s13037-023-00372-x

**Published:** 2023-07-26

**Authors:** Selim Zaghbib, Ahmed Saadi, Hamza Boussaffa, Haroun Ayed, Mohamed Riadh Ben Slama

**Affiliations:** Urology department, Charles Nicolle Hospital, 1001 Boulevard du 09 Avril, Tunis, Tunisia

**Keywords:** Iatrogenic, Ureteral injury, Gynaecological surgery, Postoperative complication

## Abstract

**Background:**

Intraoperative iatrogenic ureteral injuries represent rare technical surgical complications with the potential for adverse patient outcomes, particularly when the diagnosis is delayed. Ideally, these technical complications are recognized and repaired intraoperatively. This study was designed to investigate the root causes and outcomes of missed intraoperative ureteral injuries at a tertiary urology referral centre in Tunisia.

**Methods:**

This is a retrospective cohort study in a tertiary urology referral centre in Tunis from January 1^st,^ 2015, to December 31^st,^ 2020, including all patients with iatrogenic ureteral injury, not diagnosed intraoperatively. The factors associated with the success of endoscopic treatment and those associated with the unfavourable evolution were investigated.

**Results:**

A total of 40 iatrogenic ureteral injuries were included. Gynaecological surgery was responsible for 85% of ureteral injuries, mainly during hysterectomies (55%). The symptoms were dominated by low back pain (37.5%) and pyelonephritis (25%). Endoscopic treatment was attempted in 22 cases, it was sufficient in 12 cases. Ureteral injury required surgical treatment in 24 cases, and ureteroneocystostomy was performed in 16 cases. Nephrectomy was performed in eight cases, representing 20% of injuries, including three cases as the first treatment for late-diagnosed cases with a destroyed kidney. In the analytical study, endoscopic treatment was sufficient in 50% in case of ureteral fistula versus 27% in case of ureteral stenosis (p = 0.04). Nephrectomy was performed in 10% of cases when ureteral injury was diagnosed within the first month postoperatively compared to 60% of cases when this delay exceeded one month (p = 0.004).

**Conclusion:**

Iatrogenic ureteral injuries discovered postoperatively are mostly secondary to gynaecologic surgery. Although endoscopic treatment is usually performed as a first treatment, a more aggressive surgical is often necessary, with a nephrectomy rate of 20%.

## Introduction

Iatrogenic ureteral injury is rare but difficult to manage [1]. The frequency of operative ureteral injury after abdominopelvic surgery varies depending on the series studied [1]; McCarus et al. reported an incidence of 1% in 2.5 million abdominopelvic surgeries in the United States [2]. The retroperitoneal location of the ureter, combined with its close contact with abdominal and pelvic structures, can make it difficult to identify intraoperatively, leaving it vulnerable to accidental injury (ligation, coagulation, devascularisation) [3]. The consequences of these injuries are serious, including urinary obstruction or fistula, infectious complications, renal failure and even death [2]. In addition, such lesions can have a significant economic impact by increasing the length of hospitalisation and the cost of subsequent treatment. Iatrogenic injury to the ureter is, therefore ideally recognized intraoperatively, allowing immediate repair. However, most ureteral injuries are diagnosed postoperatively with variable delays and clinical presentations, making management challenging [4]. Indeed, management of missed intraoperative ureteral injuries varies widely, from endoscopic treatment allowing cicatrisation of the ureter to more aggressive surgical management ranging from repair of the affected ureter (ureteroneocystostomy, ureteroureterostomy, transureteroureterostomy, ureteral substitution…) to nephrectomy in case of impossible surgical repair or in case of a destroyed kidney [5, 6].

Thus, it seems essential for any surgeon to know the interventions at risk of intraoperative ureteral lesion to insist on preventive measures such as urinary tract drainage in certain cases, which has been reported to significantly reduce the incidence of ureteral injuries and good intraoperative exposure [2]. It is also important for all surgeons to be aware of the consequences of a peroperative lesion of the ureter and its associated morbidity, as well as the consequences of such a complication.

This study, therefore, has three aims: (1) to report interventions at risk of intraoperative ureteral injuries; (2) to investigate the epidemiological, clinical, and therapeutic characteristics of ureteral injuries undiagnosed intraoperatively in a tertiary referral urology centre; and (3) to assess the outcome of these injuries.

## Methods

To investigate the root causes and outcomes of missed intraoperative ureteral injuries, we designed a retrospective cohort study in a tertiary urology referral centre in Tunis from January 1^st,^ 2015, to December 31^st,^ 2020. We included all patients hospitalized in the department for iatrogenic ureteral injury, not diagnosed intraoperatively, and whose subsequent follow-up was performed in the same department. The collection of cases of ureteral injuries was based on medical records. Predefined data forms were filled in by the same urologist to reduce the risk of measurement bias. Data on patients (age and sex), ureteral injury circumstances (type of intervention, open or laparoscopic surgery), diagnosis (delay and clinical presentation), characteristics (location and type), therapeutic management (first treatment, endoscopic treatment, surgical treatment), and follow-up were collected. After excluding patients with missing data related to intervention, therapeutic management, or follow-up, the study included a total of 40 iatrogenic ureteral injuries.

The primary outcome measure was the success of endoscopic treatment, defined by the healing of the ureteral injury with a functional kidney after drainage of the excretory tract where the injury is located. The secondary outcome measure was an unfavourable evolution defined by the realization of a nephrectomy in case of an irreparable ureteral injury or in case of a destroyed kidney, either immediately or after one or several treatments.

Qualitative data were reported as numbers and percentages. Quantitative data were reported as means and standard deviations.

The factors associated with the success of the endoscopic treatment, on one hand, and with the unfavourable evolution of ureteral injuries, on the other hand, were investigated. The statistical tests used were Pearson’s chi-square or Fisher’s exact test for qualitative variables and Student’s t-test for quantitative variables. The significance level was set at p < 0.05. All statistical analyses were performed using IBM SPSS 21 software.

## Results

### Participants

Our study included 40 iatrogenic ureteral injuries occurring in 38 patients (two patients had bilateral ureteral injury), including three men and 35 women.

### Descriptive data

The mean age was 52 +/- 12.1 years. Gynaecological surgery was the most common source of these injuries, with 34 ureteral lesions (85%), mainly during hysterectomies for benign conditions (55%), followed by the Wertheim procedure (radical hysterectomy) (17.5%). Visceral surgery was responsible for ureteral injury in six cases (15%), mainly during colectomies (10%). The surgical approach was laparoscopic in six cases (15%). Table 1 summarizes the different procedures that caused iatrogenic ureteral injury in our series.

**Table 1:** Procedures causing iatrogenic ureteral injury

Type of surgery	Number	Percentage
**Gynaecological surgery**	**34**	**85**
Abdominal hysterectomy	15	37.5
Wertheim radical hysterectomy	7	17.5
Laparoscopic hysterectomy	3	7.5
Vaginal hysterectomy	2	5
Haemostasis hysterectomy	2	5
Caesarean section	2	5
Laparoscopic adnexectomy	2	5
Burch colposuspencion	1	2.5
**Visceral surgery**	**6**	**15**
Right colectomy	2	5
Laparoscopic sigmoidectomy	1	2.5
Proctocolectomy	1	2.5
Adhesiolysis	1	2.5
Restoration of intestinal continuity (Hartmann)	1	2.5

Postoperatively, ureteral injury was diagnosed during the first month in 30 cases (75%) with a mean delay of 13.4 days. In 25% of cases, the diagnosis was made beyond the first postoperative month. The symptoms were dominated by low back pain (37.5%), pyelonephritis (25%), and vaginal leakage of urine (22.5%). Table 2 summarizes the different circumstances of diagnosis of a missed intraoperative iatrogenic ureteral injury.

**Table 2:** Circumstances of diagnosis of an iatrogenic ureteral injury

Circumstances of diagnosis	Number	Percentage
Low back pain	15	37.5
Pyelonephritis	9	22.5
Vaginal urine leakage	8	20
Fortuitous	3	7.5
Urinoma	2	5
Infected urinoma	1	2.5
Pyelonephritis + vaginal leakage	1	2.5
Renal failure	1	2.5

**Fig. 1:**
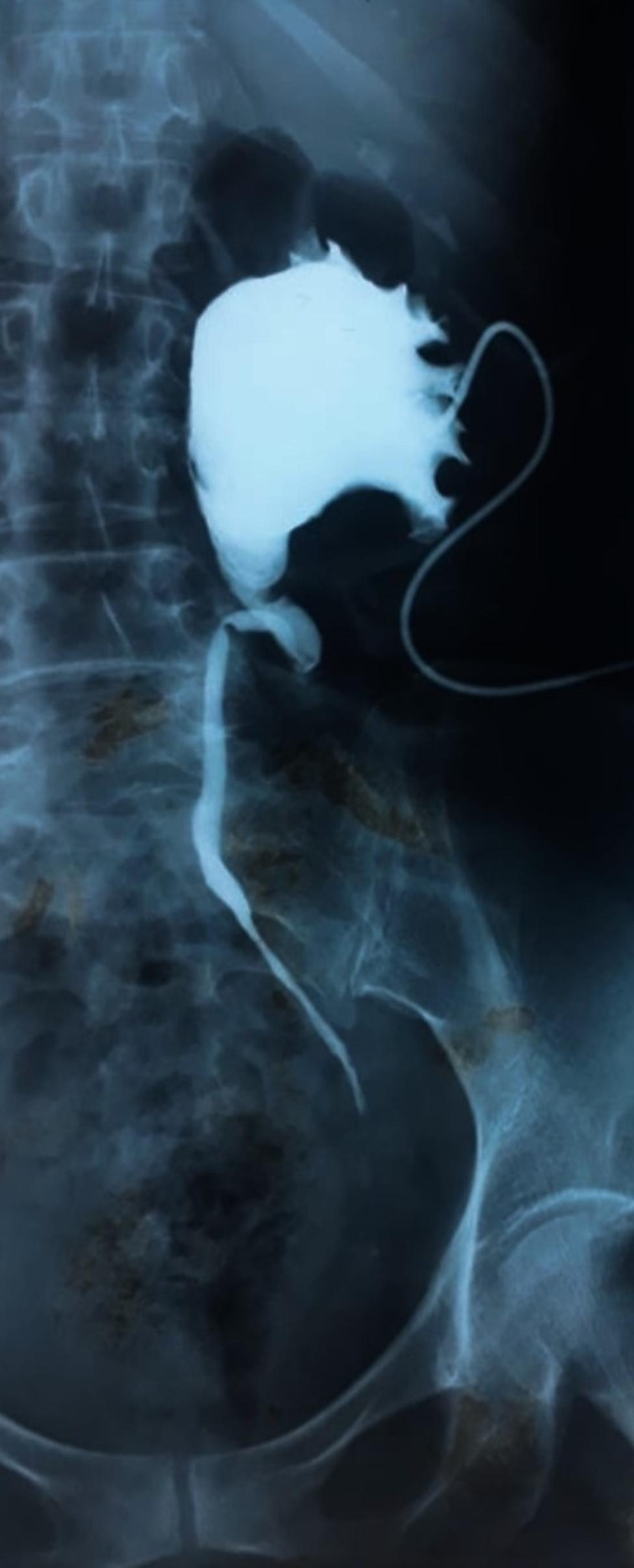
Antegrade opacification through nephrostomy catheter showing an obstruction located on the left pelvic ureter secondary to iatrogenic injury after abdominal hysterectomy

**Fig. 2:**
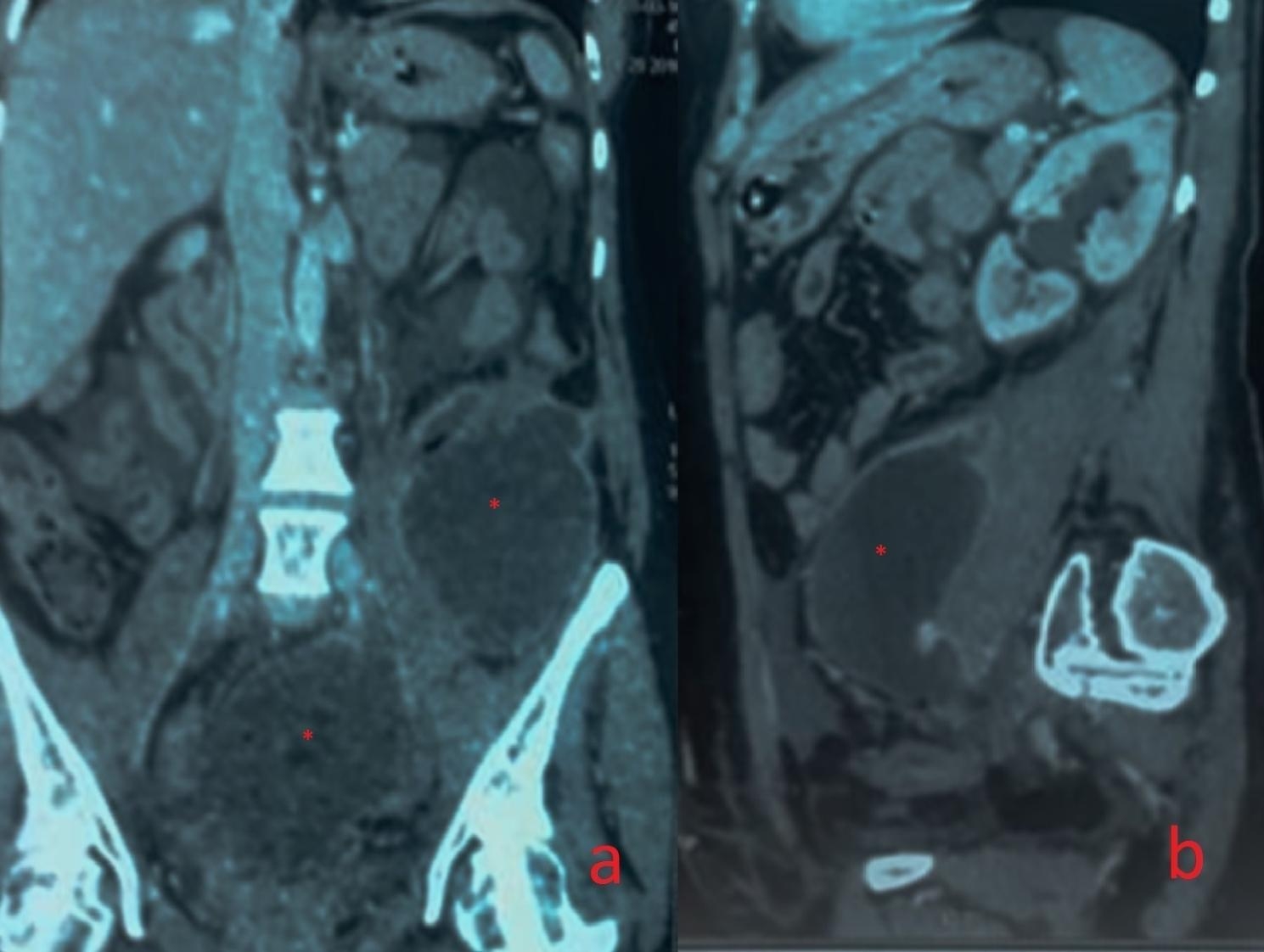
Computed tomography showing a left-retroperitoneal and pelvic urinoma (Asterisk) secondary to right iatrogenic ureteral injury (a: Frontal plane; b: Sagittal plane) after abdominal hysterectomy

Imaging (computed tomography (CT) urogram or antegrade opacification through nephrostomy catheter) localized the injury to the pelvic ureter in 80% of cases, the iliac ureter in 15%, and the lumbar ureter in 5%. The injury was located on the right side in 47.5% of cases and on the left side in 52.5%. The most frequent type of injury observed on imaging was ureteral obstruction, with no passage of contrast material in 70% of cases (Fig. 1). A ureterovaginal fistula was detected in 17.5%, and an urinoma was found in 12.5% of cases (Fig. 2). The most frequent associated lesion was a bladder injury in four cases (10%).

### Outcome data and main results

**Fig. 3:**
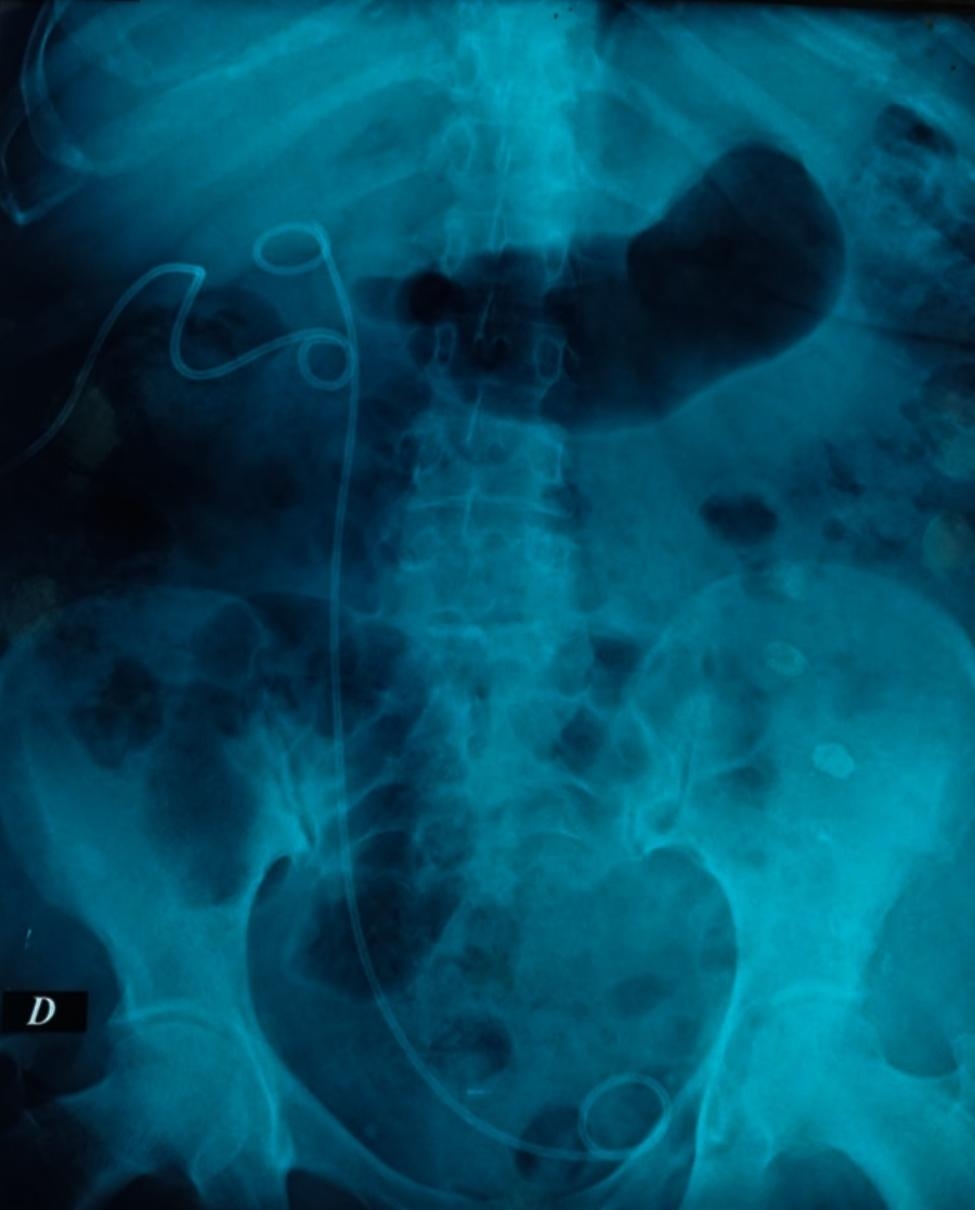
Abdominal X-ray showing double J ureteral stent placed through a nephrostomy catheter (antegrade) for a right iatrogenic ureteral injury

The first-line treatment was an attempt to insert a JJ stent (retrograde insertion) in 18 cases, with success in 50% of cases. A percutaneous nephrostomy catheter was placed at diagnosis in 15 cases with no passage of contrast product on control opacification in 10 cases (subsequent surgical treatment). In the other cases, antegrade opacification showed spontaneous healing in one case, and antegrade ureteric stenting was attempted in four cases (successful in two cases) (Fig. 3). Altogether, endoscopic treatment was attempted in 22 cases; it was sufficient in 12 cases (54.5%).

Iatrogenic ureteral injury required surgical treatment in 24 cases (60%). One patient underwent surgical repair on the first postoperative day; the section of a thread ligating the ureter was sufficient. Ureteroneocystostomy was performed in 16 cases (40%), three of which were performed as first treatment. When ureteroneocystostomy was performed in the second instance, a nephrostomy tube was placed, and the average intervention delay was three months. In two cases, a vesico-psoas hitch was realized. Necrosis of the ureter had occurred in one case after a ureteroneocystostomy (without vesico-psoas hitch) requiring a nephrectomy.

Nephrectomy was performed in eight cases, representing 20% of ureteral lesions, including three cases as the first treatment for late-diagnosed ureteral injury with a destroyed kidney and after one or more treatments in the other cases. Table 3 summarizes the management of missed intraoperative iatrogenic ureteral injury in our series.

**Table 3:** Iatrogenic ureteral injury management

First Treatment (n)	Outcome (n)	Second treatment (n)	Third treatment (n)
Nephrostomy + ureteral stenting attempt (4)	Success (2) Failure (2)	Ureteroneocystostomy (1) Nephrectomy (1)	--
Nephrostomy without ureteral stenting attempt (11)	Spontaneous healing (1) Functional kidney (8) Non-functional kidney (2)	Ureteroneocystostomy (8) Nephrectomy (2)	Nephrectomy for ureteral necrosis (1)
Ureteral stenting attempt (18)	Success (10) Failure (8)	Nephrostomy (7) Nephrectomy (1)	Ureteroneocystostomy (5) Lost to follow-up (1) Deceased before treatment (1)
Thread Sect. (1)	Success	-	-
Ureteroneocystostomy (3)	Success	-	-
Nephrectomy (3)	-	-	-

In the analytical study, the type of injury on imaging was associated with the outcome of endoscopic treatment: In the 22 cases where it was attempted, endoscopic treatment was sufficient in 50% in case of ureteral fistula versus 27% in case of ureteral obstruction (p = 0.04). The rate of nephrectomy (unfavourable evolution) was associated with the interval between the procedure causing the ureteral injury and diagnosis: Nephrectomy was performed in 10% of cases when the injury was diagnosed within the first month postoperatively compared to 60% of cases when this delay exceeded one month (p = 0.004).

## Discussion

Our study, including 40 cases of missed iatrogenic intraoperative ureteral injuries, showed that gynaecological surgery was responsible for 85% of those injuries, mainly during hysterectomies. Iatrogenic ureteral injury was diagnosed during the first month in most cases, and the symptoms were dominated by low back pain, pyelonephritis, and vaginal leakage of urine. Endoscopic treatment was sufficient in 12 out of 22 cases. Ureteral injury required surgical treatment in 24 cases, and ureteroneocystostomy was performed in 16 cases. Nephrectomy was performed in eight cases, representing 20% of ureteral injuries. Endoscopic treatment had a higher probability of succeeding in ureteral fistula when compared to ureteral stenosis, and the nephrectomy rate was higher in lately diagnosed intraoperative ureteral injuries.

In addition to the retrospective nature of the data collection, the main limitation of our study is the exclusion of a certain number of patients because of missing data concerning the intervention or the follow-up. Ureteral lesions diagnosed intraoperatively were not included because the purpose of our study was to show the morbidity of these lesions in case of postoperative diagnosis to emphasize the importance of prevention and immediate diagnosis in high-risk surgery.

The epidemiology of ureteral injuries has evolved considerably in recent decades [7]. Indeed, iatrogenic injuries are now the main cause of ureteral wounds (98%) [8]. The estimated frequency of intraoperative ureteral injuries during abdominopelvic surgery varies greatly depending on the series analysed [1]. However, the incidence of ureteral lesions was increased approximately fivefold in prospective studies if a search for a potential injury intraoperatively after intravenous injection of indigo carmine was performed [9]. In gynaecological surgery, the incidence of iatrogenic ureteral injuries ranges from 0.013 to 1.8% [10]. This surgical discipline alone accounts for 47–55% of all ureteral injuries reported in the literature. In our series, gynaecologic surgery was the cause of ureteral lesions in 80% of cases [10]. This frequency also depends on the type of surgery performed: Hysterectomy is the procedure most frequently responsible for these lesions, with a reported rate that ranges from 0.3 to 1.8% [6]. Whether the surgery is abdominal or vaginal, the ureter is always at risk [8]. Radical hysterectomies with lymphadenectomy (Wertheim type) are the greatest providers of ureteral injuries: 10 to 30% of cases [4]. In our study, hysterectomy for a benign condition was the most frequent cause, followed by the Wertheim procedure. Moreover, laparoscopic surgery does not spare this organ; In a Norwegian study over 11 years, the rate of ureteric injury after laparoscopic hysterectomy was 1% (33.4% of ureteric injuries after hysterectomy) [6]. However, the incidence of intraoperative ureteral injuries remains more frequent in open surgery than in laparoscopy [6], as demonstrated by our results, where laparoscopy was responsible for 15% of those injuries.

Besides, visceral surgery is responsible for 15 to 23% of ureteral injuries (15% in this study) [6]. Colorectal surgery, particularly abdominoperineal resection, and sigmoidectomy exposes the ureter to iatrogenic injury, especially during intestinal dissection. The risk of uretero-colic fistulisation is more frequent than uretero-intestinal fistulisation [11]. Vascular surgery accounts for 4 to 10% of ureteral lesions [10]. A few cases have also been reported after orthopaedic surgery (spine) [10]. We did not find any ureteral injury secondary to vascular or orthopaedic surgery.

Different types of iatrogenic ureteral injuries are identified: obstruction (by ischemia, ligation, or crushing) or fistulisation (in the vagina, uterus, and peritoneum, through the scar or the drainage system). The injury mechanisms found were ligation (wires or clips), sectioning, crushing, resection and denudation by dissection altering the vascularization [8]. In laparoscopy, coagulation injuries damaging the ureteral vascularization are the most common [12].

Ideally, iatrogenic ureteral injury is diagnosed intraoperatively and treated immediately. Unfortunately, 50–70% of ureteral injuries are not diagnosed intraoperatively [1, 12, 13]. Our study focused exclusively on postoperative diagnosed ureteral injuries, and the diagnosis was made in the first month after surgery in 75% of cases.

The most frequent diagnostic circumstances are low back pain and fever related to pyelonephritis in most cases or infected urinoma. Low back pain may be present depending on the nature of the injury and whether the ureter is occluded or has fistulated to the peritoneal cavity, retroperitoneal space, or to vagina in women [6].

The CT urography allows the diagnosis to be made. Urogram locates the injury by showing an arrest of the contrast material in case of obstruction or a leakage in case of fistula. In the latter case, the dilatation of the excretory cavities may be absent [14].

The treatment choice is based on the topography, extension, time between the onset of the intraoperative ureteral injury and diagnosis (early or late), mechanism, and patient comorbidities [4, 15]. Deferred surgery (three to six months after the initial lesion) to permit the resolve of inflammatory processes and the restoration of tissue integrity is the standard management of delayed diagnosis ureteral lesions. However, more recently, initial endourological treatment has been shown to substantially reduce morbidity, reduce re-operation rates and promote spontaneous recovery [16].

Thus, the endoscopic approach is often preferred as the first-line treatment. Retrograde ureteral catheterization represents, for several authors, the first initiative, as is the case in our centre. In case of failure, the percutaneous approach with anterograde catheterization of the excretory tract is used [16, 17]. However, antegrade upper tract urinary diversion can be proposed as a first-line procedure offering the possibility of an antegrade placement of a JJ stent if deemed feasible after opacification [8].

No consensus has been established on the duration of the JJ stent, but several authors suggested that the stent could be safely removed between two and six weeks [18]. In the literature, endourological treatment was successful in 33 to 64% of cases [16]. In this study, the success rate was 54.2%.

If endoscopic treatment is impossible or fails, surgical treatment becomes indispensable. Usually, a waiting period of 6 weeks to 3 months was suggested before reconstructive surgery. This period is necessary for inflammation, fibrosis, adhesions, tissue oedema, and anatomical distortions to disappear [18]. Other authors have reported comparable results for immediate post-diagnosis reconstruction versus delayed repair [18]. These results do not allow a definitive conclusion to be drawn, and the timing of ureter repair must be decided according to the patient and the surgeon’s habits [18]. In our centre, this delay was, on average, three months.

Anterograde pyelogram using a nephrostomy catheter is the reference imaging method to localize the level of the injury, and to adapt the surgical treatment. In the case of a functional kidney, the surgical treatment depends on the location of the ureteral injury.

Ureteroneocystostomy is preferred when the lesion is distal (< 3-5 cm) above the ureterovesical junction) and less than 2 cm [8]. Indeed, above these limits, a bladder elongation to allow tension-free reimplantation had to be associated using the vesico-psoas hitch technique or the Boari-tubularized bladder flap [8].

In case of small (2–3 cm) defects of the mid-ureter and upper ureter, a ureteroureterostomy can be performed, with the placement of a double-J catheter protecting the anastomosis [8].

In case of an extensive defect, a trans-ureterostomy (terminal-lateral anastomosis between the damaged and the healthy ureter) was a feasible option at the cost of risks of damage to the healthy ureter [10].

Finally, regardless of the level of injury, when the loss of substance is too great, ureteroileoplasty remains a reference technique of last resort, given its morbidity [19]. Renal auto transplantation may also be an option, requires an extensive discussion with the patient about the potential complications [20].

## Conclusion

Our study has shown that iatrogenic ureteral injuries discovered postoperatively are mostly secondary to gynaecologic surgery. Although endo-urological treatment is usually performed as a first treatment, more aggressive surgical treatment is often necessary. The location of the injury dictates surgical management, and ureteroneocystostomy for distal injuries is the most commonly employed technique. Missed iatrogenic intraoperative ureteral injury can lead to nephrectomy (20% in the present study), hence the interest in prevention by good exposure and a preoperative location of the ureters in cases at risk.

## Data Availability

The datasets used and/or analysed in this study are available from the corresponding author upon reasonable request.
